# Substance accumulation of a wetland plant, *Leersia japonica*, during senescence in the Yihe and Shuhe River Basin, North China

**DOI:** 10.3389/fpls.2022.996587

**Published:** 2022-10-13

**Authors:** Xiuyi Yang, Guanqun Wang, Shutong Lei, Zongfeng Li, Bo Zeng

**Affiliations:** ^1^ College of Agriculture and Forestry Science/Library; Linyi University, Linyi, China; ^2^ Key Laboratory of Eco-Environment in the Three Gorges Reservoir Region, Ministry of Education, Faculty of Life Science, Southwest University, Chongqing, China

**Keywords:** alcohol dehydrogenase, *Leersia japonica*, membrane stability, root vitality, substance transportation

## Abstract

*Leersia japonica* is a perennial Gramineae grass that is dominant in shallow wetlands of the Yihe and Shuhe River Basin, North China. Previous studies have shown that *L. japonica* recovers early (March), tillers strongly, and has an excellent ability to purify sewage in spring. This early revival might play a vital role in water purification function; however, whether the plant benefits from the physiological activities during senescence remains unclear. Therefore, in this study, an experiment was conducted during the winter of 2016 and in the following spring. Morphology (height, biomass, root morphology), physiology (root vitality, malondialdehyde [MDA], superoxide dismutase [SOD]), substance contents (soluble sugar, soluble protein) and substance transportation (activity of enzymes for transportation and energy supply) were determined during weeks 0, 2, 4, 6, and 8 of the senescence stage (October 11, 2016); as well as substance contents and bud increments during days 0,7, 14, 21, 31 and 41 of the revival period (February 22, 2017). The results revealed that (1) the root biomass of *L. japonica* increased significantly during senescence, even after the leaves withered. (2) The root diameter of *L. japonica* decreased significantly, while root weight per volume and root superficial area per volume increased significantly during senescence. The root vitality was relatively stable in winter, especially for root absorption area per volume. (3) No significant difference was observed in membrane stability of stems, rhizomes and roots of *L. japonica* in winter, with the MDA content remaining stable and SOD activity increasing significantly during senescence. (4) The soluble sugar content of all tissues of *L. japonica* increased sharply during senescence; while it decreased significantly in spring, especially for buds. (5) The enzymes for substance metabolism responded differently, with activities of H^+^-ATPase and pyruvate decarboxylase (PDC) decreasing, and alcohol dehydrogenase (ADH) increasing. Therefore, *L. japonica* has active morphological adaptation of roots, physiological regulation, and massive substance accumulation during senescence stage. The special life-history trait ensures *L. japonica* survival in winter and revival in early spring, which makes it being an excellent plant for purifying sewage in spring.

## Introduction

Wetlands are among the most vibrant ecosystems in the world (Kumwimba et al., 2021) and are a functioning landscape (Qu et al., 2022) and water purification system (Wang et al., 2022). At present, the purification function of wetlands has received extensive attention, and a number of constructed wetlands have been built to deal with sewage (Jia et al., 2021; Wang et al., 2021). As a key factor of wetlands, plants are always selected by evaluating their purifying efficiency (Qiao et al., 2021). Generally, plants purify polluted water in two ways. Some plants absorb pollutants (nitrogen, phosphorus, organic matter and heavy metals) through their roots from the habitat and accumulate them in their shoots (Liu et al., 2021; Yan et al., 2021). Other plants decompose pollutants (such as nitrogen and phosphorus) with rhizome-sphere microorganisms (Wang et al., 2022).

The water purification efficiency of plants changes clearly with the seasons. In growth seasons, most wetlands will purify polluted water *via* absorption and decomposition of plants (Li et al., 2021; Wang et al., 2022). However, when plants begin to wither during senescence, wetlands will noticeably reduce or lose their purification efficiency (Wang et al., 2021). The death of wetland plants will not only reduce the physiological activity of water purification of wetlands because of energy shortage with photosynthetic efficiency decline (Huang et al., 2010) but also increase the risk of secondary pollution in the following spring (Li, 2009). Water pollution of wetlands is not likely to become immediately worse in winter due to low decomposition activity (Zhan et al., 2021); however, when the weather turns warmer, water pollution incidents occur frequently (Petraglia et al., 2018). Spring is the rush hour of blooming (Vernet et al., 2021). Wetland plants that recover early in spring are likely to have an advantage in being selected as water-purifying plants.

*Leersia japonica* is a perennial Gramineae grass that is dominant in shallow wetlands of the Yihe and Shuhe River Basin, North China. It has a long growth period (from March to December) and strong tillering ability (**Supplementary Figure 1**) and is likely to be an alternative plant for water purification. However, it is usually treated as a weed in paddy fields (Tian et al., 2018), and little attention has been given to its water purification mechanisms. In a previous study, we found that *L. japonica* significantly reduced the content of chemical oxygen consumption (COD) and total nitrogen (TN), two main indices for evaluating water pollution, during spring (unpublished, **Supplementary Figure 2**). The recovery of perennial plants is indispensable to substance accumulation in the senescence stage and membranes over winter (Bao et al., 2022). However, the physiological response of *L. japonica* to senescence, which leads to early revival, remains unclear.

It is imperative to precisely estimate the biomass components of *L. japonica* to evaluate its senescence processes. Plants usually initiate their anti-stress mechanism when they progress into the senescence stage (Thomas, 2013). In addition to maintaining membrane stability (evaluated by malondialdehyde (MDA) content and antioxidant enzyme activity (i.e., superoxide dismutase [SOD]) (Zhao et al., 2021), plants accumulate substances and undergo morphological and physiological adaptations to deal with senescence stress (Thomas, 2013). When temperatures drop, some macro-molecules in plant cells are hydrolyzed and their soluble sugar content increases. Soluble sugar is a vital substance used by plants to tolerate chilling stress, and it is significantly accumulated during the winter (Wang et al., 2019). Generally, nutrient accumulation in plants during the senescence stage originates from shoot recycling and absorption (Havé et al., 2017). Previous studies demonstrated that the root absorption ability of plants is related to their root morphology (i.e., specific surface area) (Suzuki et al., 2003), as well as energy status (i.e., H^+^-ATPase activity) (Miao et al., 2022). This ability is usually evaluated by root activity, which is reflected by the specific absorption area (i.e., absorption area per root volume) (Han et al., 2016). Anaerobic metabolism involves two key enzymes, pyruvate decarboxylase (PDC) and alcohol dehydrogenase (ADH), which catalyze the breakdown of pyruvate to the end product, ethanol, through an intermediate, acetaldehyde (Frazierab et al., 2020), and cytochrome c oxidase (CCO), the terminal electron acceptor in the mitochondrial electron transport chain that is directly responsible for more than 95% of oxygen metabolism (Phan et al., 2016). Therefore, understanding the physiological activities of *L. japonica* during senescence is vital for studying the revival and water purification functions in the following spring.

Generally, a perennial plant develops a unique strategy to survive in the senescence stage, including morphological and physiological responses, as well as substance accumulation (Roach and Smith, 2020). In this study, morphological (height, biomass, root morphology), physiological (root vitality, membrane stability), substance accumulation (soluble sugar, soluble protein), and substance transportation (enzymes for supplying energy) characteristic of *L. japonica* during senescence, as well as bud increments and substance content in the revival period were investigated. We hypothesized that (i) *L. japonica* responds actively to senescence in terms of its morphological and physiological characteristics, and we asked the following questions: Which morphological characters responded effectively to senescence? Does *L. japonica* maintain stable membrane during senescence and tolerate cold stress successfully in winter? (ii) Substance accumulation occurs *via* root absorption and transportation during the senescence stage, and we asked which adaptive traits of *L. japonica* have been produced to meet the energy needs of material absorption and transportation during senescence?

## Materials and methods

### Plant materials

*Leersia japonica* (Makino) Honda is a perennial Gramineae grass that is distributed in the shallow wetlands of North, South and Southwest China. It is a dominant species in the wetlands of the Yihe and Shuhe River Basin, North China (35.1183N, 118.2840E).

Seedlings were collected from a carefully-selected community (May 30, 2016) where *L. japonica* was the only dominant species and where sampling and cultivation management was convenient (flat terrain, stable water level). Seven hundred seedlings of similar height (10–15 cm tall) were collected and planted in soft plastic pots (Ø 26 × 21 cm) using the habitat topsoil from a *L japonica*-dominant community. The soil was loam with litter sand and nutrients were determined as follows: soil organic matter content 2.16 ± 0.23%, total nitrogen 0.10 ± 0.01%, alkali-hydrolysable nitrogen 215.40 ± 27.86 mg/kg, rapidly available phosphorus 73.60 ± 5.05 mg/kg, and rapidly available potassium 94.80 ± 5.53 mg/kg.

Three seedlings were planted in each pot to produce sufficient tillers for determination. Three pots were placed in a polyethylene bucket (Ø 50 × 50 cm) with a water depth of 30–40 cm. Sixty buckets of *L. japonica* were cultivated in total to investigate the physiological characteristics of senescence and recovery period (30 buckets for each). All buckets were placed in the wetland (water level was 20 cm) near the *L. japonica* population.

At the beginning of cultivation, water was changed every 3 days to remove fermented soil products. After seedling recovery, the water level was controlled by precipitation or water addition and maintained 20 cm above the soil. Daily management, such as weed and pest elimination, was conducted to ensure the healthy growth of *L. japonica.*

### Experimental design

**Figure 1:**
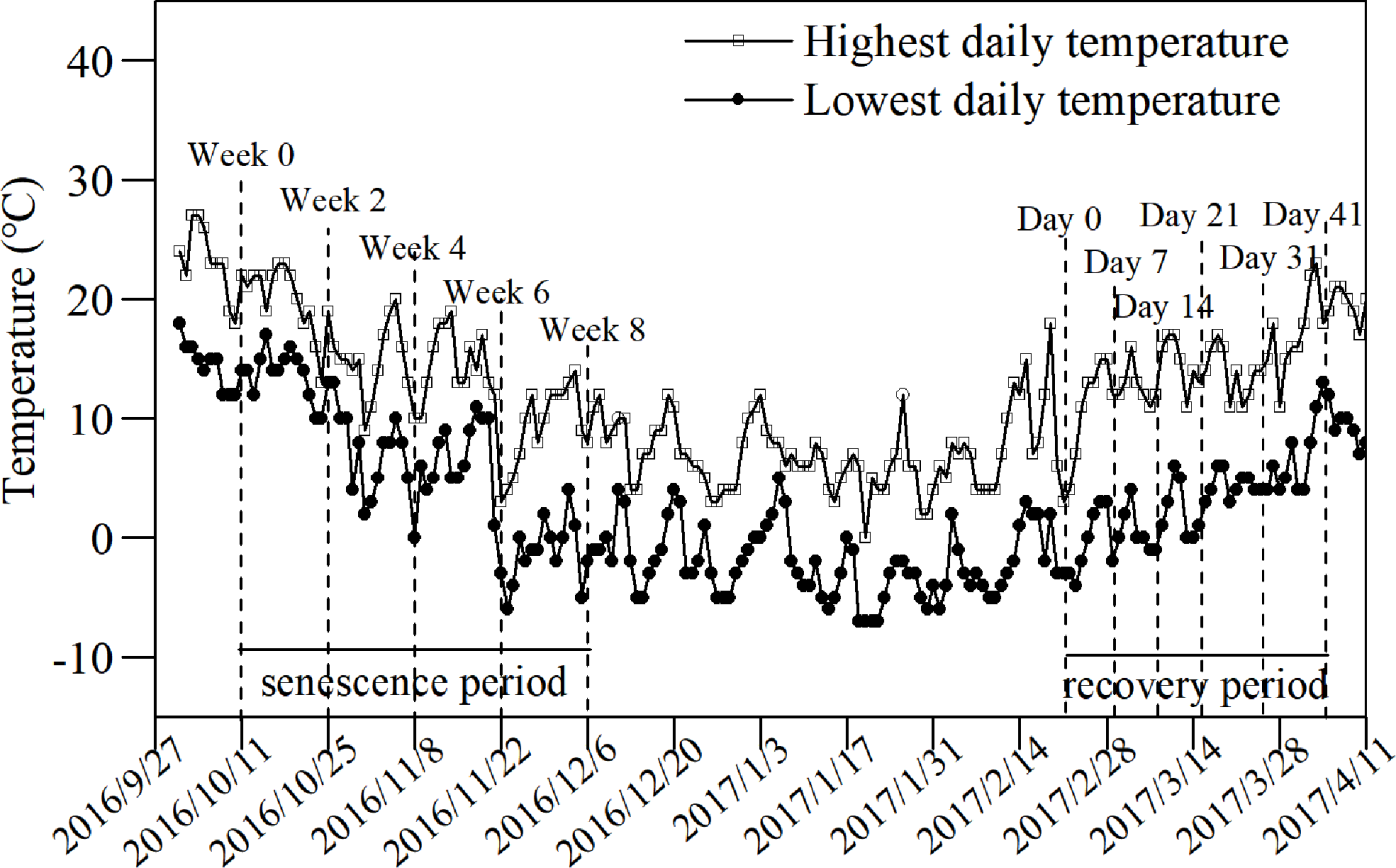
Changes in daily temperatures during the decline and revival stages of *L. japonica*. Lines with blank squares and filled circles indicate the highest and lowest daily temperatures, respectively. The dashed lines indicate the sampling date.

The senescence phase of the experiment began when roughly half of the leaves had withered (October 11, 2016; **Figure 1**). The water level was precisely controlled to ensure experiment consistency. The experiment lasted for 8 weeks. A bucket was selected randomly as 1 replicate with 5 replicates each time. Samplings were conducted at the beginning of the senescence stage and every 2 weeks afterward until week 8. The lowest daily temperature was 0°C in week 4 and remained below 0°C for most of the experiment after week 6 (**Figure 1**).

To investigate the recovery process of *L. japonica*, the increments in bud height were recorded in the following spring. Three pots of *L. japonica* were selected after the shoots withered and 10 buds in each pot were marked. The heights of all marked buds were recorded from the soil surface on days 0, 7, 14, 21, 31, and 41 during the recovery experiment when the lowest and highest daily temperatures were just below 0°C and 10°C, respectively (**Figure 1**).

### Sampling

Plastic pots were kneaded slightly for soil softening before sampling and scoured with tap water to remove the soil from the roots of *L. japonica.* The roots were washed carefully, controlling the flushing position of seedlings and water flow to avoid root damage. During this process, seedling integrity was ensured by preventing potential damage to the root system. Live leaves, stems (5 cm long, cut from the base of the stem), representative roots (1 cluster for a pot, ~ 3 g fresh weight) and rhizomes (~ 5 g fresh weight) were collected for detecting the substance content and enzymatic activity of *L. japonica* during senescence.

### Measurements

To investigate the senescence process, single tiller biomass (fresh weight) and its components (i.e., leaves, stems, rhizomes and roots) were weighted after carefully washing and removing withered leaves and stems. To save sampling time and reduce the difference in the number of tillers among replicates, three tillers of *L. japonica* in each bucket were collected. The average fresh weight value was recorded as the single tiller biomass. Before washing, plant height was measured from the soil surface, and chlorophyll content was determined with a living chlorophyll analyzer (SPAD). After sampling, root vitality was immediately determined using the dyeing method described by Zhang and Li (2016), and root morphology was determined with a rhizome scanner (WinRhizo STD 4800).

The soluble protein content was determined following the Coomassie brilliant blue color method, as described by Zhang and Li (2016). Plant samples (0.2–0.5 g fresh weight) were ground with 1–2 mL water. The solution was diluted to 10 mL after 3 extractions and centrifuged at 5000 r/min at 4°C for 20 min. The absorbance was measured at 595 nm using a UV-visible spectrophotometer. The soluble sugar content was determined using the anthrone-sulfuric acid method, as described by Li and He (2021). Soluble sugar was extracted from 0.2 g samples (fresh weight) with 80% (v/v) aqueous ethanol in a water bath at 80°C for 40 min. The solution was diluted to 25 mL after 3 extractions. One mL solution (0.2 mL extracted solution, 0.8 mL water) was mixed with 5 mL of anthrone-sulfuric acid, as described by Zhang and Li (2016). The absorbance was measured at 626 nm using a UV-visible spectrophotometer.

Enzymatic activities were evaluated by enzyme-linked immunosorbent assay (ELISA) on a Rayto-6100 ELISA analyzer (Ji and Iwasaki, 2022). Enzymes were extracted with phosphate buffer solution (50 mM, pH 7.4) from plant samples (0.2–0.5 g fresh weight). The solution was diluted to 10 mL after 3 extractions and centrifuged at 3000 r/min at 4°C for 20 min. Plant enzymes (i.e., α-amylase, H^+^-ATPase, CCO, PDC, and ADH) were combined with a purified relative enzyme antibody and made into an antibody-antigen-enzyme-antibody complex. After complete washing, 2 color developing agents, tettillerhylbenzidine and hypothalamic regulatory peptides, were successively added to the complex. The absorbance was measured at 450 nm on a Rayto-6100 ELISA analyzer. The enzymatic activities were calculated based on the standard curves.

### Statistical analyses

Statistical analyses were performed using SPSS v19.0 (SPSS Inc., IL, USA). A one-way analysis of variance (ANOVA) was conducted to compare the means of plant morphology, substance contents, and enzymatic activities during the senescence stage. Before conducting ANOVA, the homogeneity of variances was tested. If the homogeneity of variance was assumed, Duncan’s multiple-range test was used to test whether plant heights, soluble sugar contents, and H+-ATPase activities differed between sampling periods. Data were presented as mean ± standard error (SE), which were obtained from five independent replicates. Figures were created using Origin v9.0 (OriginLab, MA, USA).

## Results

### Temperature recorded during the senescence and revival stages

The ambient temperature was recorded during the experiment period (**Figure 1**). Samplings were conducted at the beginning of the senescence stage and every 2 weeks afterward until week 8. With certain fluctuations, the temperature gradually decreased in both its highest and lowest temperatures. By week 8, the temperature had increased slightly and rose beyond the temperature of week 6. The temperature clearly exhibited a fluctuating increase at the beginning of the revival stage. The highest temperature rose from ~5°C at the beginning to ~20°C by day 41, while the lowest temperature rose from −5°C at the beginning to ~10°C by day 41.

### Morphological and physiological response of *L. japonica* to senescence

#### (1) Dynamics of plant height and relative chlorophyll contents during senescence

**Figure 2:**
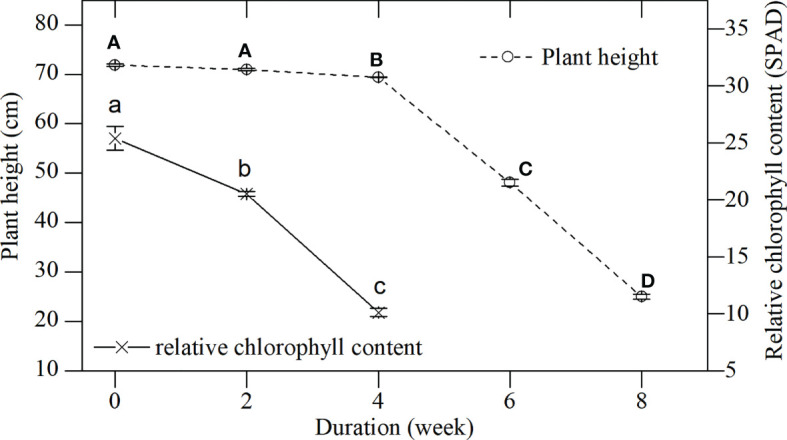
Plant height and relative chlorophyll content of *L. japonica* during the senescence stage. Data are presented as mean ± standard error (SE). Each batch consisted of five replicates. The height and chlorophyll content of each replicate were obtained from 10 stems and 30 leaves. Different capital and small letters indicate significant differences (*p* < 0.05) in plant height and relative chlorophyll content of *L. japonica*, respectively.

Plant height (live shoots) and the chlorophyll content of *L. japonica* decreased significantly during senescence (plant height, *p* < 0.01; relative chlorophyll content, *p* < 0.01, **Figure 2**). Plant height decreased slightly, while chlorophyll content decreased sharply during the first 4 weeks. All leaves died back between weeks 4 and 6. Plant height decreased due to the withered leaves and was 34.79% the height of the plant by week 8 when compared with week 0 (25.01 ± 0.48 cm vs 71.89 ± 0.26 cm).

#### (2) Dynamics of biomass and components during senescence

Single tiller biomass and components of *L. japonica* were weighted during senescence (**Table 1**). No significant differences were detected in single tiller biomass between weeks, while significant differences were detected in biomass components (single tiller biomass, *p* = 0.426; leaves, *p* < 0.01; stems, *p* < 0.01; roots, *p* < 0.01). Shoot biomass (leaves and stems) decreased significantly. The biomass of leaves and stems by week 4 was significantly lower when compared with week 0, being 14.12% and 80.80% of the latter, respectively. Moreover, stem biomass further decreased, reaching up to 40.80% by week 8. Root biomass significantly increased during senescence, however, significant differences were not observed until week 6.

**Table 1:** Biomass and components of *L. japonica* during the senescence stage.

Sampling period	Single tiller biomass (g)	Biomass components (g)			
		Leaf	Stem	Rhizome	Root
Week 0	3.51 ± 0.15ns	0.34 ± 0.02a	1.25 ± 0.06a	0.10 ± 0.01ns	1.83 ± 0.14b
Week 2	3.63 ± 0.13ns	0.14 ± 0.01b	1.23 ± 0.05a	0.10 ± 0.00 ns	2.16 ± 0.09b
Week 4	3.36 ± 0.16ns	0.05 ± 0.01c	1.01 ± 0.06b	0.08 ± 0.01ns	2.21 ± 0.15b
Week 6	3.75 ± 0.09ns		0.82 ± 0.05c	0.09± 0.05ns	2.85 ± 0.13a
Week 8	3.56 ± 0.17ns		0.51 ± 0.03d	0.07 ± 0.01ns	2.98 ± 0.17a

Data are presented as the mean ± SE. Each batch consisted of 5 replicates. Different letters indicate significant differences (p < 0.05). ns indicates no significance.

#### (3) Dynamics of root morphology and root vitality during senescence

The root morphology and vitality of *L. japonica* varied during senescence (**Table 2**). Most

**Table 2:** Dynamics of root morphology and root vitality of *L. japonica* during the senescence stage.

Sampling period	Root diameter(mm)	Root weight per volume(g/cm^3^)	Root superficial area per volume (cm^2^/cm^3^)	Root length ratio (%)					Root vitality	
				I	II	III	IV	V	Root absorbing area per volume (m^2^/cm^3^)	Root active absorbing area (%)
Week 0	0.56 ± 0.03a	1.02 ± 0.076c	72.30 ± 3.23b	87.18 ± 0.58b	9.56 ± 0.41b	1.89 ± 0.13b	0.66 ± 0.11a	0.72 ± 0.20a	0.13 ± 0.01a	45.29 ± 2.04a
Week 2	0.57 ± 0.02a	1.12 ± 0.05bc	71.40 ± 2.57b	84.46 ± 0.55c	11.93 ± 0.36a	2.52 ± 0.15a	0.79 ± 0.09a	0.30 ± 0.04a	0.09 ± 0.01b	33.02 ± 0.22c
Week 4	0.44 ± 0.02c	1.22 ± 0.05b	92.22 ± 4.61a	91.60 ± 0.50a	7.58 ± 0.41b	0.69 ± 0.10b	0.12 ± 0.03b	0.02 ± 0.01b	0.15 ± 0.01a	36.55 ± 0.51bc
Week 6	0.42 ± 0.01c	1.48 ± 0.03a	95.93 ± 1.76a	91.23 ± 1.14a	8.31 ± 1.12b	0.43 ± 0.06cd	0.03 ± 0.01b	0.00 ± 0.00b	0.13 ± 0.01a	40.07 ± 2.36ab
Week 8	0.50 ± 0.01b	1.46 ± 0.05a	80.01 ± 1.74b	92.51 ± 2.27a	7.29 ± 0.98b	0.19 ± 0.04d	0.01 ± 0.00b	0.00 ± 0.00b	0.15 ± 0.01a	34.56 ± 2.97bc

Data are presented as the mean ± SE. Each batch consisted of 5 replicates. Numbers I–V indicate root levels (i.e., < 1 mm, 1–2 mm, 2–3 mm, 3–4 mm, and > 4 mm basal diameter, respectively). Different letters indicate significant differences (p < 0.05).

*L. japonica* roots were fine, with ~90% of root lengths ranking as I (d < 1 mm). The root morphology varied significantly during the senescence stage, and significant differences were detected in root diameter, root weight per volume, root superficial area per volume, and root length ratio between weeks. The root weight per volume of *L. japonica* increased significantly during senescence, while that in weeks 4, 6, and 8 was significantly higher when compared to week 0, reaching a 145.10% increase in week 6. Root superficial area per volume and root length ratio exhibited an increasing trend during senescence.

The root vitality remained stable during senescence and no differences were detected the in root absorbing area per volume between weeks, except for in week 2. However, the root vitality absorbing area decreased significantly; in weeks 2, 4, and 8 it was significantly lower than in week 0. All of the root morphological indices of root active absorbing area at week 4 were significantly different when compared with week 0, with the root weigh per volume, root superficial area per volume, and root length ratio all being higher than the latter; no differences were detected for root absorption area per volume.

#### (4) Membrane stability of *L. japonica* to senescence

**Figure 3:**
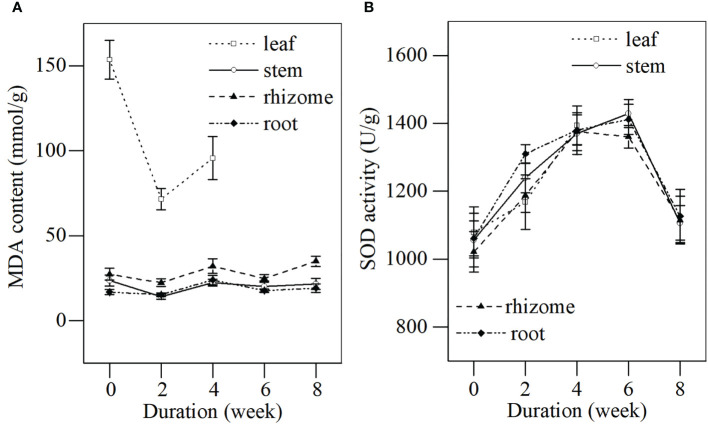
MDA content and SOD activity in different tissues of *L. japonica* during the senescence stage. Data are presented as mean ± SE. Each batch consisted of five replicates. **(A)** MDA content, **(B)** SOD activity.

The MDA content and SOD activity in different tissues of *L. japonica* were determined during the senescence stage (**Figure 3**). The MDA content in the leaves of *L. japonica* decreased significantly at a faster rate (from 153.72 ± 11.46 to 71.49 ± 6.36 mmol/g), while other tissues fluctuated at a slower rate (stems, from 14.04 ± 1.43 to 23.61 ± 3.21 mmol/g; rhizomes, from 22.29 ± 2.19 to 34.88 ± 3.10 mmol/g; roots, from 15.26 ± 1.14 to 23.96 ± 2.76 mmol/g). SOD activities in all tissues of *L. japonica* increased significantly for the first 4 weeks and increased by a similar amount, which was ~30% of the amplification when compared with week 0. The SOD activities in stems, rhizomes, and roots decreased to their initial levels by week 6.

### Substance accumulation and transportation of *L. japonica*

#### (1) Dynamics of soluble sugar and protein content during the senescence and the revival stages

**Figure 4:**
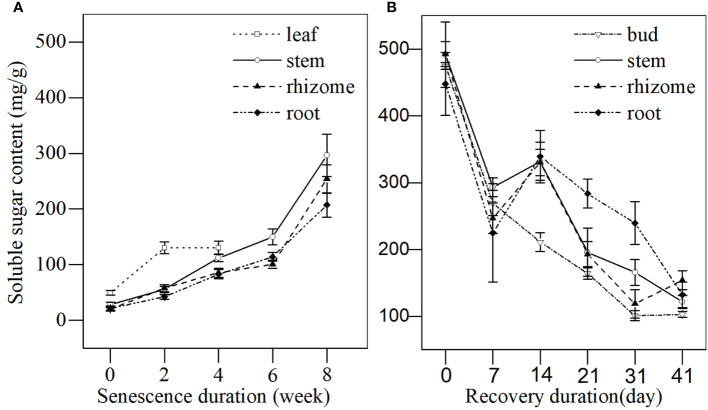
The soluble sugar content in different tissues of *L. japonica* during senescence and revival. Data are presented as mean ± SE. Each batch consisted of five replicates. **(A)** Soluble sugar content during senescence, **(B)** Soluble sugar content during revival.

As shown in **Figure 4A**, when plants entered the senescence stage, the soluble sugar content in leaves increased during the first 2 weeks and peaked by week 4 before leaves withered completely. The soluble sugar content in stems, rhizomes, and roots consistently increased throughout the senescence period. By week 8, stems, rhizomes, and roots stored a large amount of soluble sugar, reaching up to 1045.63%, 1221.12%, and 966.32% higher when compared with week 0, respectively. The soluble sugar content increased continuously over winter, reaching up to ~500 mg/g in all the tissues (**Figure 4B**). When plants entered spring, the soluble sugar content in buds, stems, rhizomes, and roots decreased significantly and remained stable until days 21 and 31 (**Figure 1**). The soluble sugar content by days 31 and 41 was significantly lower that at day 0 in all tissues. The soluble sugar content of buds decreased faster than that of the other tissues, especially for the roots. This indicated that *L. japonica* revives in mid-March and that buds consume more soluble sugar for revival.

**Figure 5:**
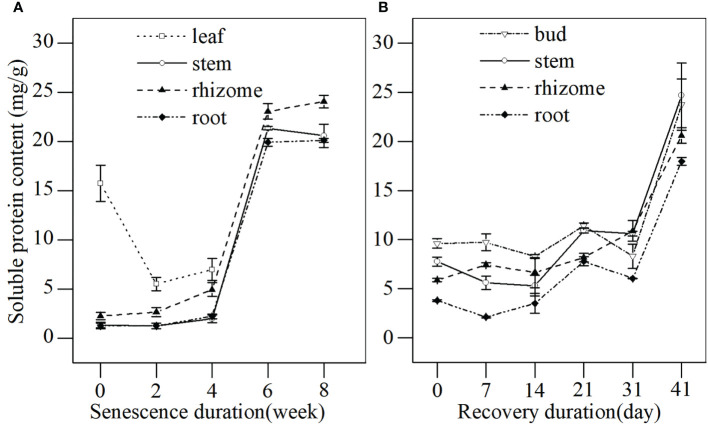
The soluble protein content in different tissues of *L. japonica* during senescence and the revival. Data are presented as mean ± SE. Each batch consisted of five replicates. **(A)** Soluble protein content during senescence, **(B)** Soluble protein content during revival.

The soluble protein content reflects the metabolic level and aging process of plants. The soluble protein content in different tissues of *L. japonica* was detected during the senescence stage (**Figure 5A**). A decrease in the soluble protein of leaves was detected before the leaves withered completely, while the soluble protein content in stems, rhizomes, and roots increased throughout the senescence period. The soluble protein in stems, rhizomes, and roots increased slightly for the first 4 weeks, then increased sharply between weeks 4–6 by 995.38%, 366.80%, and 801.81%, respectively, when compared with week 0. The protein content in buds, stems and rhizomes of *L. japonica* increased gradually, and a significant difference occurred by days 21, 21 and 31, respectively, when compared with day 0 (**Figure 5B**).

#### (2) Dynamics of H^+^-ATPase and α-amylase activities of different tissues during senescence

**Figure 6:**
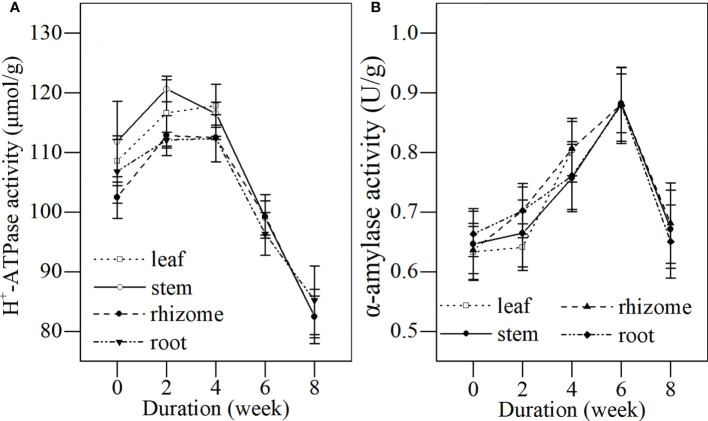
Dynamics of H^+^-ATPase and α-amylase activity in different tissues of *L. japonica* during the senescence stage. Data are presented as mean ± SE. Each batch consisted of five replicates, which had two repetitions in parallel. **(A)** H^+^-ATPase activity, **(B)** α-amylase activity.

During the first 4 weeks of the senescence stage, H^+^-ATPase activities were detected at higher levels in the leaves, stems, rhizomes, and roots (**Figure 6A**). This indicated that all tissues of *L. japonica* could actively conduct substance transportation and components. The H^+^-ATPase activities in stems, rhizomes, and roots of *L. japonica* significantly declined after week 4, indicating a reduced energy supply for substance transportation as leaves withered.

The α-amylase activities in all tissues of *L. japonica* increased during the first 4 weeks of the senescence stage (**Figure 6B**), indicating that the hydrolysis of starch was active at a high level. The α-amylase activities in stems, rhizomes, and roots of *L. japonica* decreased significantly after week 6, and the enzymatic activities seemed to be inhibited as temperatures dropped during the senescence stage.

#### (3) Dynamics of CCO, PDC, and ADH activities of different tissues during senescence

**Figure 7:**
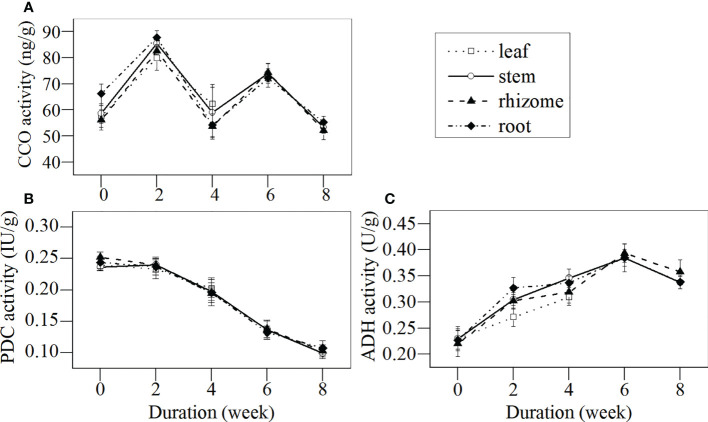
Dynamics of CCO, PDC, and ADH activities in different tissues of *L. japonica* during the senescence stage. Data are presented as mean ± SE. Each batch consisted of five replicates, which had two repetitions in parallel. **(A)** CCO activity, **(B)** PDC activity, **(C)** ADH activity.

Key enzymes of aerobic respiration (i.e., CCO) and anaerobic respiration (i.e., PDC and ADH) in different tissues of *L. japonica* during the senescence stage were investigated (**Figure 7**). The CCO activities in all tissues fluctuated throughout the senescence period and no significant differences were detected, except in the roots, which decreased. The CCO activities in all tissues by week 2 were significantly higher when compared with week 0, while no significant differences were detected between weeks 4 and 0. The PDC activities in the leaves, stems, rhizomes, and roots decreased, while the ADH activities in all tissues increased significantly over time.

### Dynamics of bud increments during the recovery stage

**Figure 8:**
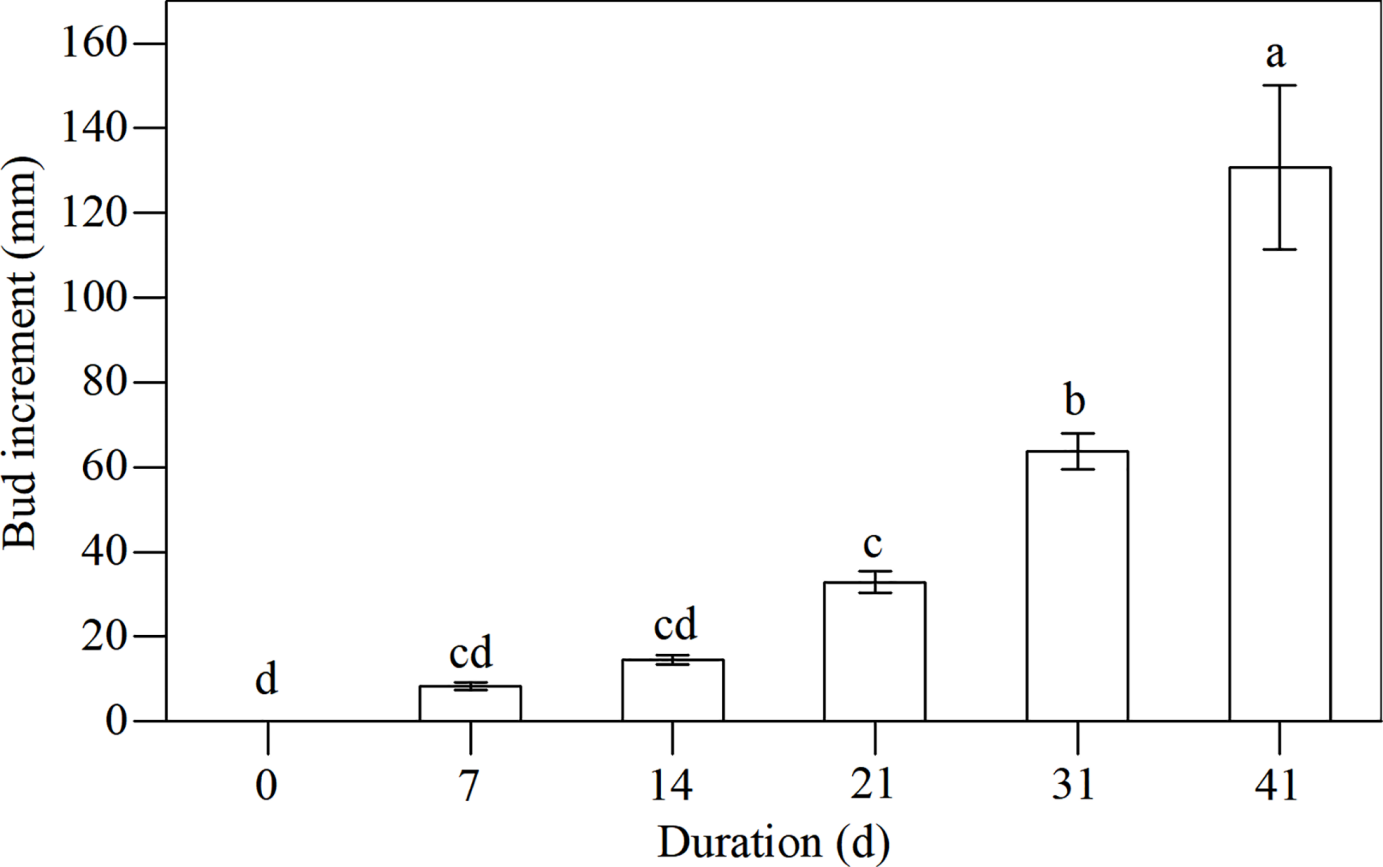
Dynamics of bud increments of *L. japonica* during the recovery stage. Data are presented as mean ± SE. Each batch consisted of five replicates. Different letters a-d indicate significant differences (p < 0.05) in bud increment of L. japonica.

An important criterion for the recovery stage is bud growth. When conditions became favorable (i.e., mild temperatures), the plants prepared to grow. **Figure 8** shows the increment of bud height at days 7, 14, 21, 31, and 41, indicating a robust growth rate. By day 21 of the recovery experiment (March 15), bud heights increased significantly with bud increments being greater than 20 mm.

## Discussion

Bud heights of *L. japonica* increased significantly by day 21 of the revival (**Figure 8**), indicating that *L. japonica* is a species that revives in the early spring (mid-March) when the lowest daily temperatures were still below 0°C (**Figure 1**). The soluble sugar content increased sharply during senescence and decreased significantly, especially for the buds (**Figure 4**). This indicates that soluble sugar plays a vital role in overwintering and subsequent recovery, as reported by previous research (Bao et al., 2022). It is more difficult for perennial wetland plants to revive in early spring than terrestrial ones (Che and Zhao, 2004; Tian et al., 2013) because their dormant buds suffer a long-lasting cold habitat, including a frozen and ice-water mixture (Guittar et al., 2016). The early recovery of wetland plants must be sufficiently prepared, including physiological adaptation and substance accumulation, during the senescence stage.

### Morphological and physiological responses of *L. japonica* to senescence

In this study, *L. japonica* gradually withered from early October to early December, and retained live stems longer than 20 cm when the daily temperature dropped below 0°C (**Figures 1**, **2**). This indicated that *L. japonica* had a late-starting and long-lasting senescence, which allowed *L. japonica* to be sufficiently prepared for overwintering. First, *L. japonica* recycles substances during senescence. No significant differences were observed in the biomass of a single tiller during the senescence stage, although shoots gradually withered (**Table 1**, **Figure 2**). This may be because the increase in root biomass offsets the loss of withered shoots. The increased root weight per volume indicated that *L. japonica* accumulated substances *via* recycling during the senescence stage (**Table 2**). Unlike our findings, the biomass of many wetland plants decreased from summer to winter (Lee et al., 2013). Next, *L. japonica* adjusts root morphological characteristics during senescence. In this study, the length proportion of fine roots (d < 1 mm) increased significantly, and the root superficial area per volume increased significantly (**Table 2**). This indicated that *L. japonica* enhanced its root surface, which comes into direct contact with minerals, and could conduct absorption *via* the apoplast pathway (Munns et al., 2019). The rapid response of roots to low temperature in winter was also reported in previous studies (D'Imperio et al., 2018). Therefore, the active and excellent morphological responses contributed to the survival of *L. japonica* during senescence.

No significant increase was detected in the MDA content of the stems, rhizomes, and roots of *L. japonica* (**Figure 3A**), although plants suffered from frozen stress during senescence in December. This was likely due to the increased antioxidant enzymatic activity. The SOD activity of the stems, rhizomes, and roots of *L. japonica* increased significantly, followed by a decrease when frozen (**Figure 3B**). The stable membrane and increased SOD activity under low-temperature stress were also reported in previous studies (Li et al., 2008; Peng et al., 2019). In addition, root absorption area per volume and root active absorbing area remained stable after a short period of adjustment (**Table 2**), indicating that *L. japonica* maintained vigorous root vitality and was likely to absorb substances for physiological activities, even when shoots withered in winter. The similar results were also reported in the cold-tolerant wetland plant *Oenanthe javanica* (Zuo et al., 2017). Therefore, the excellent physiological response contributed to the survival of *L. japonica* during senescence.

In a word, *L. japonica* in the Yihe and Shuhe River Basin developed morphological and physiological response for survival during senescence. It not only actively adjusted root morphological characteristics for maintaining vigorous root vitality (**Table 2**), but also maintained a stable membrane (**Figure 3**) to ensure the survival of stems, rhizomes and roots of *L. japonica* overwinter (**Figures 3**, **4**). What’s more, *L. japonica* accumulate substance to support physiological activities overwintering and recovery, with less loss of biomass during senescence (**Table 1**).

### Substance accumulation and transportation of *L. japonica* during senescence

It is believed that substance accumulation guarantees overwintering success and the revival of plants (Wang et al., 2019). Plants usually accumulate soluble sugar to tolerate cold stress in winter (Brown et al., 2020), as well as accumulate soluble protein to support enzyme synthesis, dormant bud occurrence, and subsequent revival in spring (Brown et al., 2020). In this study, the substance accumulation of *L. japonica* was reflected in the increased biomass (**Table 1**) and the increased contents of soluble sugar and protein of the roots, even after the leaves withered by week 6 (**Figures 4A**, **5A**). However, the substance accumulation of *L. japonica* during senescence suffers from several difficulties, especially from energy deficiency under cold-stress conditions and from its leaves being withered. Moreover, the energy required to maintain physiological activities during the senescence stage depends on anaerobic metabolism after leaves die.

In this study, the H^+^-ATPase activity in the stems, rhizomes, and roots decreased significantly during the senescence stage (**Figure 6**). This indicated that *L. japonica* faced some difficulties in ATP synthesis and ion transmembrane transportation; thus, it was difficult for it to recycle nutrients from their shoots and to absorb minerals from its habitat. Furthermore, the activity of PDC, a key enzyme for decomposing pyruvic acid to acetaldehyde (Eknikom et al., 2022), in the roots of *L. japonica* decreased significantly (**Figure 7B**), which affected the energy supply of *L. japonica* from anaerobic metabolism. Luckily, the activity of ADH, another key enzyme of anaerobic metabolism (Bhatla, 2018), increased significantly during senescence(**Figure 7C**), thereby reducing acetaldehyde to ethylalcohol, the terminal step of fermentation. The increased ADH activity of *L. japonica* ensured the smooth operation of fermentation. Thus, *L. japonica* is clearly an excellent species that accumulates sufficient substances during the senescence stage and is well prepared for revival in the following season.

In summary, the plant biomass, root morphology, contents of soluble sugar and protein, activities of H^+^-ATPase and aerobic and anaerobic metabolic enzymes in different parts (i.e., roots, stems, leaves, and rhizomes) during the senescence stage were investigated in this study to explore the metabolic physiology of *L. japonica*. The active morphological adaptation of roots and physiological regulation ensured that *L. japonica* respond to the low temperature stress effectively during senescence and thus provided the necessary substances guarantee for its early revival in the following spring. The findings of this study will serve as a theoretical basis for better understanding this species’ physiological characteristics during the senescence stage.

## Data availability statement

The original contributions presented in the study are included in the article/**Supplementary Material**. Further inquiries can be directed to the corresponding author.

## Author contributions

SL and BZ conceived and designed the experiments. XY and GW analyzed the data. SL, XY, and ZL wrote the manuscript. All authors reviewed and approved of the manuscript.

## Funding

This study was supported by the Science and Technology Fund Project of Shandong Provincial Department of Agriculture and Rural Affairs (2019LY005) and the National Natural Science Foundation of China (Nos 32202601, 31500371).

## Acknowledgments

We would like to thank Accdon (www.accdon.com) for its linguistic assistance during the preparation of this manuscript.

## Conflict of interest

The authors declare that the research was conducted in the absence of any commercial or financial relationships that could be construed as a potential conflict of interest.

## Publisher’s note

All claims expressed in this article are solely those of the authors and do not necessarily represent those of their affiliated organizations, or those of the publisher, the editors and the reviewers. Any product that may be evaluated in this article, or claim that may be made by its manufacturer, is not guaranteed or endorsed by the publisher.
