# Supplementary figures and images for: Substance accumulation of a wetland plant, Leersia japonica, during senescence in the Yihe and Shuhe River Basin, North China
Source: Front Plant Sci. 2022 Oct 13;13:996587. doi: 10.3389/fpls.2022.996587 (PMC9608780; doi:10.3389/fpls.2022.996587)

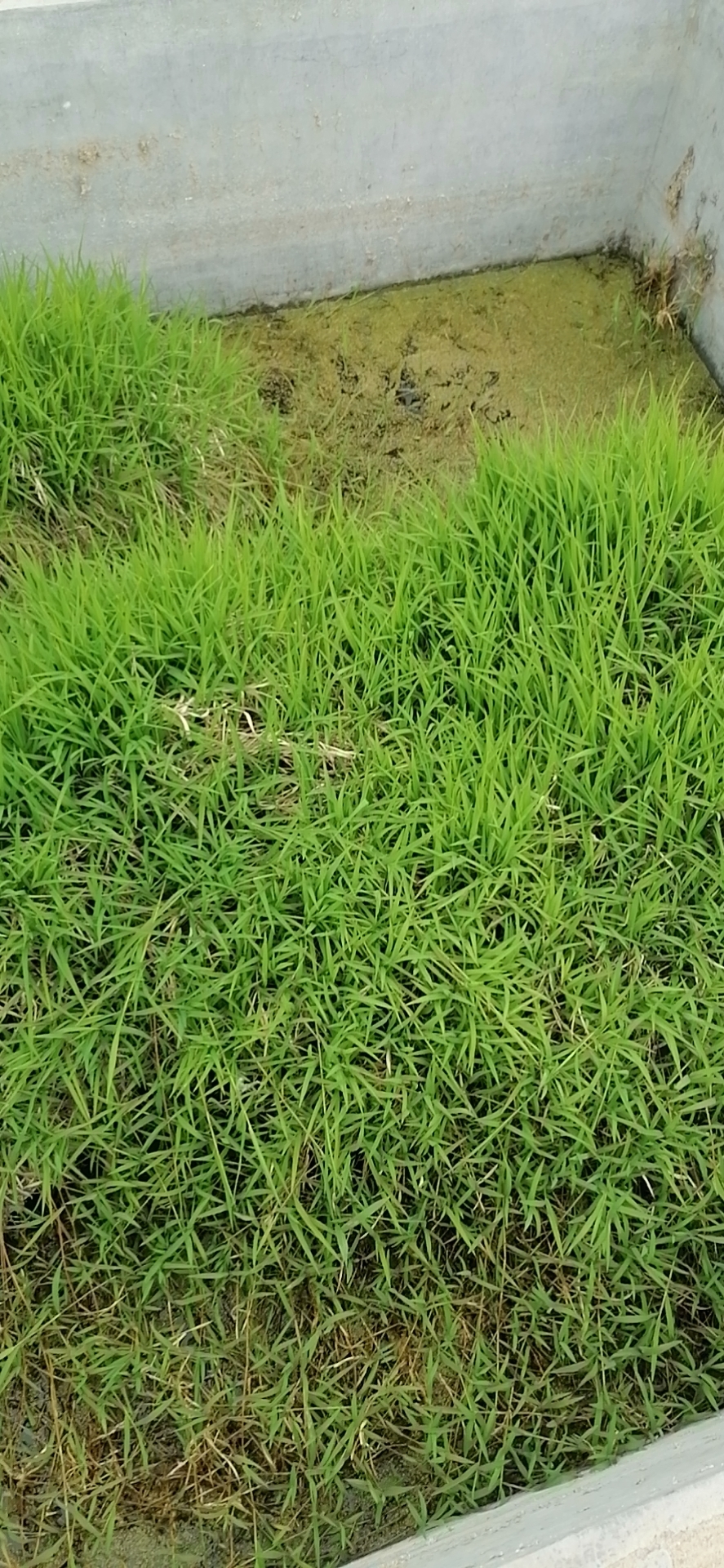

Supplement: Supplementary file 1 [file Image_1.jpeg]
